# SlMDH3 Interacts with Autophagy Receptor Protein SlATI1 and Positively Regulates Tomato Heat Tolerance

**DOI:** 10.3390/ijms25137000

**Published:** 2024-06-26

**Authors:** Sitian Wang, Li Zhang, Linyang Zhang, Kang Yong, Tao Chen, Lijun Cao, Minghui Lu

**Affiliations:** 1 College of Horticulture, Northwest A&F University, Yangling 712100, China; 15191021199@163.com (S.W.); zl236426@163.com (L.Z.); 17869725925@163.com (L.Z.); yongkang@nwafu.edu.cn (K.Y.); chengtao61@163.com (T.C.); 2 Department of Biology, Duke University, Durham, NC 27708, USA; lc353@duke.edu; 3 Howard Hughes Medical Institute, Duke University, Durham, NC 27708, USA

**Keywords:** malate dehydrogenase, heat stress, autophagy, ATI1, tomato

## Abstract

Autophagy, a highly conserved protein degradation system, plays an important role in protecting cells from adverse environmental conditions. ATG8-INTERACTING PROTEIN1 (ATI1) acts as an autophagy receptor, but its functional mechanisms in plants’ heat stress tolerance remain unclear. In this study, using LC-MS/MS, we identified malate dehydrogenase (SlMDH3) as a SlATI1-interacting protein. Further studies showed that heat stress induced the expression of *SlMDH3* and SlMDH3 co-localized with SlATI1 under both 22 °C and 42 °C heat treatment conditions. Moreover, silencing of *SlMDH3* increased the sensitivity of tomato to heat stress, as evidenced by exacerbated degradation of chlorophyll; accumulation of MDA, H_2_O_2_, and dead cells; increased relative conductivity; and inhibition of stress-related gene expression. Conversely, overexpression of *SlMDH3* improved tomato’s heat tolerance, leading to opposite effects on physiological indicators and gene expression compared to *SlMDH3* silencing. Taken together, our study suggests that SlMDH3 interacts with SlATI1 and positively regulates tomato heat tolerance.

## 1. Introduction

Global warming increases the frequency of extreme high temperatures [1], with heat stress emerging as one of the most severe abiotic stresses that impede plants’ growth, development, and reproduction. Heat stress induces protein misfolding, denaturation, oxidation, and aggregation, disturbing crucial biological processes within plant cells [2]. Unlike mobile organisms, plants cannot escape the adverse effects of high temperatures. Instead, they have evolved a complex set of mechanisms at the physiological, biochemical, and molecular levels to mitigate heat stress [3], with the efficient degradation of aggregated proteins playing a vital role in this process [4].

Protein degradation serves essential functions during plants’ development and in their responses to environmental stimuli, primarily occurring via two pathways: the ubiquitin–proteasome system (UPS) and autophagy. While the UPS plays a dominant role in the degradation of soluble and short-life proteins, autophagy is responsible for the aggregated and long-life proteins, non-protein substances such as nucleic acids and lipid bodies, and even entire organelles [5]. Autophagy is executed by sequential autophagy-related proteins (ATGs), through which the cellular cargoes are sequestered into autophagosome, a double-membrane engulfing vesicle, and then transported to the vacuole for degradation [6,7]. The involvement of autophagy in plants’ responses to heat stress has attracted many investigations. For example, in Arabidopsis, autophagy contributes to heat stress memory by NBR1-mediated degradation of HSP90.1 and ROF1 [8], while in tomato, the heat tolerance responses were compromised in autophagy-suppressed plants [9].

Autophagy is a selective degradation mechanism targeting specific cellular components or proteins under different cellular conditions [10]. The recognition of specific cargo in the autophagy pathway relies on cargo receptors, which directly or indirectly identify and tether cargos into autophagosomes by interacting with ATG8, the core protein of autophagy, through the Atg8-interacting motif (AIM) or LC3-interacting region (LIR) [11]. Studies have shown that plant ATG8s and ATG8-interacting proteins are associated with plants’ growth and responses to hormones and abiotic stresses [11,12,13,14,15]. Multiple autophagy receptors have been identified in plants, such as NBR1, DSK2A/B, MAPR5, ORM1, ATI1, and ATI2 [16]. During carbon starvation in Arabidopsis, ATG8-interacting protein1 (AtATI1) localizes to the endoplasmic reticulum (ER) and plastids to form ATI-ER and ATI-PS bodies, which are then transported to the central vacuole for selective turnover of specific proteins [17,18]. In addition, the expression of *AtATI1* in Arabidopsis can rescue the salt-hypersensitive phenotype displayed by the knockdown double mutant of *ati1ati2* [18].

Malate dehydrogenase (MDH) serves as a pivotal enzyme in the tricarboxylic acid (TCA) cycle and C4 photosynthesis, facilitating the reversible conversion between malic acid and oxaloacetic acid (OAA) [19]. MDH plays a key role in plant growth and development by participating in multiple cellular physiological activities, including mitochondrial energy metabolism, the malate–aspartate shuttle system, reactive oxygen species metabolism, and disease resistance [20]. Furthermore, MDH significantly contributes to plants’ responses to environmental stresses by regulating the cellular redox balance, photosynthesis, and antioxidative defense mechanisms [21,22]. However, the involvement of MDH in plants’ heat tolerance remains unclear.

Tomato (*Solanum lycopersicum* L.) is a widely cultivated vegetable crop around the world; however, it is highly sensitive to high temperatures, resulting in plant wilting, pollen abortion, and reduced fruit yield and quality [23]. In this study, a tomato SlATI1-interacting protein, tomato malate dehydrogenase 3 (SlMDH3 Solyc02g063490.2), was identified via the liquid chromatography–tandem mass spectrometry (LC-MS/MS) technique. Subsequent studies demonstrated that silencing *SlMDH3* rendered tomato plants more sensitive to heat stress, while overexpressing *SlMDH3* enhanced tomato’s heat tolerance. Our findings shed light on the role of *SlMDH3* in improving the heat tolerance of tomato plants, contributing to the understanding of the mechanisms underlying plant resilience to heat stress and the development of tomato varieties with higher heat tolerances.

## 2. Results

### 2.1. SlMDH3 Interacts with SlATI1

**Figure 1:**
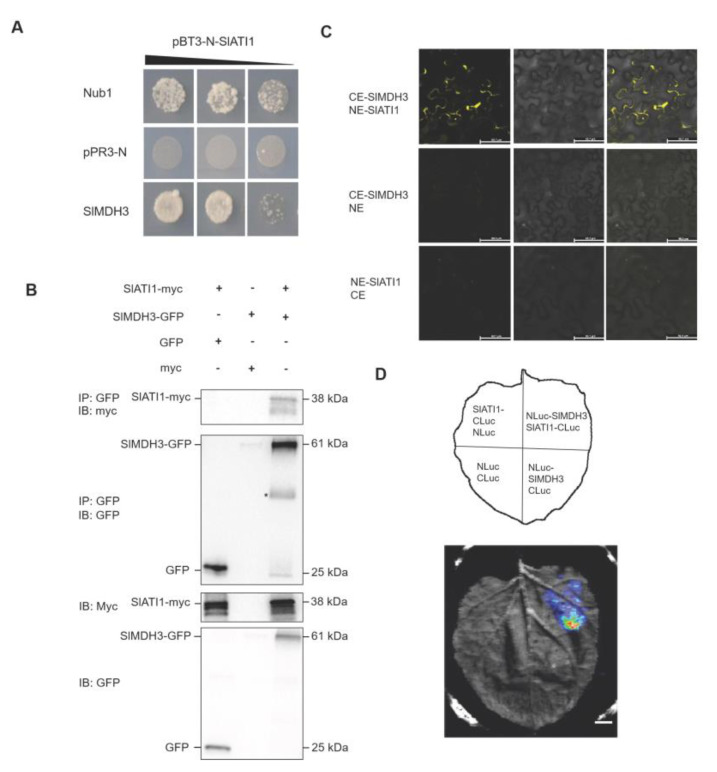
SlMDH3 interacts with SlATI1. (**A**) Interaction of SlATI1 and SlMDH3 in Y2H assay. Nub1, positive control; pPR3-N, negative control. (**B**) Co-IP assay of in vivo interaction between SlMDH3 and SlATI1. Constructs of SlMDH3-GFP and SlATI1-Myc were co-transfected in *N. benthamiana* leaves. Protein samples of inputs and outputs were immunoprecipitated with anti-GFP or anti-Myc antibodies.* represents unknown protein. (**C**) Interactions of SlMDH3 and SlATI1 in bimolecular fluorescence complementation (BiFC) assays. SlATI1 and SlMDH3 were fused to N-terminal or C-terminal portions of yellow fluorescent protein (NE or CE) and then transiently co-expressed after agroinfiltration in *N. benthamiana* and incubated at 22 °C for 3 d under light conditions. Construct pair CYFP-SlMDH3 + nYFP was co-transfected as negative control. Microscope’s magnification: 10 × 40. (**D**) Split luciferase (Luc) assay of SlATI1-SlMDH3 interaction. SlMDH3 and SlATI1 were fused to CLuc and NLuc, respectively, and were transiently co-expressed in *N. benthamiana* leaves. Construct pairs SlATI1-CLuc + NLuc, NLuc-SlMDH3+ CLuc, and NLuc + CLuc were co-transfected as negative controls.

To identify the interacting proteins of SlATI1 in plants’ heat tolerance, we employed immunoprecipitation coupled with LC-MS/MS, using SlATI1 as the bait to screen for its interacting proteins. Six candidate proteins were identified (Table S1), among which MDH was chosen for further validation due to its known role in plant tolerance to abiotic stress [24]. The finding was further confirmed using Y2H and Co-IP assays in vitro. Specifically, the yeast cells co-transformed with SlMDH3, and SlATI1 could grow on a SD/-Leu/-Trp/-His/-Ade medium (Figure 1A), whereas growth was not observed in other combinations, and SlMDH3-GFP was coimmunoprecipitated with SlATI1-Myc in vitro (Figure 1B). In addition, we detected that SlATI1 interacts with SlMDH3 in vivo through BiFC and split luciferase assay. Strong fluorescent (Figure 1C) and luminescence (Figure 1D) signals were detected when SlATI1 and SlMDH3 were co-expressed in tobacco (*Nicotiana benthamiana*) leaves. These results demonstrated interactions between SlATI1 and SlMDH3.

### 2.2. SlMDH3 Responds to Heat Treatment and Co-Localizes with SlATI1 under Heat Stress

**Figure 2:**
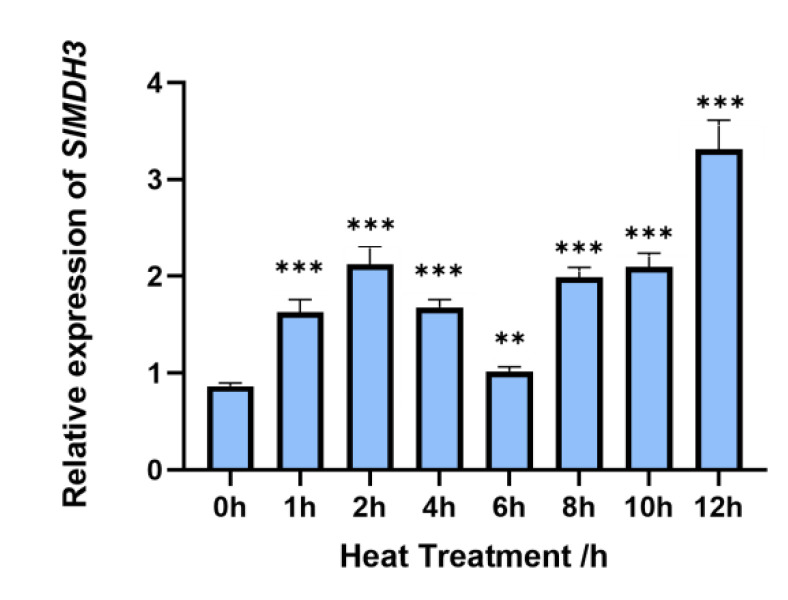
Analysis of *SlMDH3* expression during heat stress in tomato (cv. Micro-Tom). Plants were subjected to heat treatment at 42 °C with three biological replications, each consisting of three plants at 5–6 leaf stage. **, *p* < 0.01; ***, *p* < 0.001.

To investigate whether *SlMDH3* responds to heat stress, tomato seedlings were exposed to heat stress at 42 °C, and the leaves were sampled at different timepoints after the treatment. The *SlMDH3* expression was significantly increased at each timepoint of heat treatment compared to 0 h (Figure 2), which is consistent with the findings of Imran [25]. These data suggest that *SlMDH3* does indeed respond to heat stress.

**Figure 3:**
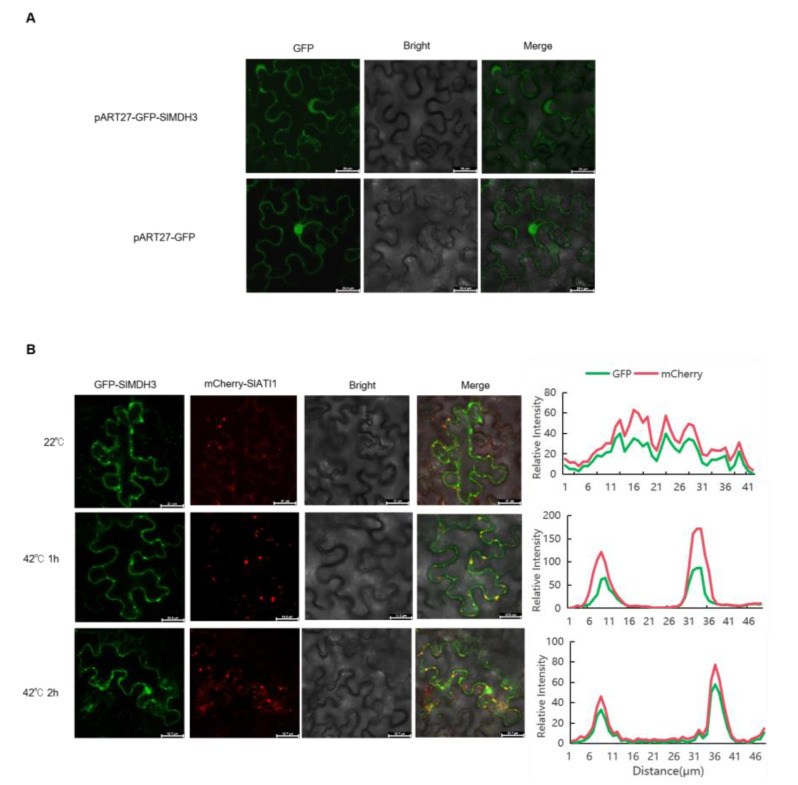
SlMDH3 co-localizes with SlATI1. (**A**) Subcellular localization of SlMDH3 in cytoplasm. (**B**) SlATI1 co-localizes with SlMDH3 under normal and high temperatures. All constructs were infiltrated into *N. benthamiana* leaves using *Agrobacterium* strain GV3101. Green, red, and yellow colors show signals of GFP-SlMDH3, mCherry-SlATI1, and their merged protein, respectively. Microscope’s magnification: 10 × 40.

Subsequently, we investigated the subcellular localization of SlMDH3 by transiently expressing it in the epidermal cells of tobacco leaves. The GFP signal was observed in the cytoplasm of leaves injected with *agrobacterium* harboring the plasmid of pART27-GFP-SlMDH3, while in leaves injected with the empty vector pART27, the signal was uniformly distributed across the entire cell (Figure 3A). Given that SlMDH3 interacts with SlATI1 and responds to heat stress, we further explored their subcellular localization under a high temperature. When pART27-GFP-SlMDH3 and pART27-mCherry-SlATI1 were transiently co-expressed in the epidermal cells of tobacco leaf, the green fluorescence of the SlMDH3:GFP fusion protein was overlapped with the red fluorescence of the SlATI1:mCherry fusion protein under a normal temperature, while a more pronounced overlap was observed under heat stress (Figure 3B). These results indicate that SlMDH3 co-localizes with SlATI1.

### 2.3. SlMDH3-Silenced Plants Display Decreased Tolerance to Heat Stress

In order to investigate the involvement of *SlMDH3* in plants’ heat stress tolerance, we generated *SlMDH3*-silenced tomato plants (TRV2:SlMDH3) by using virus-induced gene silencing (VIGS) technology. Upon observing the photo-bleaching phenotype in the leaves of positive control plants TRV2:SlPDS, we measured the expression level of *SlMDH3* in TRV2:SlMDH3 and the control plants TRV2:00. Plants with a silencing efficiency over 70% (Figure S1) were chosen for further analysis.

**Figure 4:**
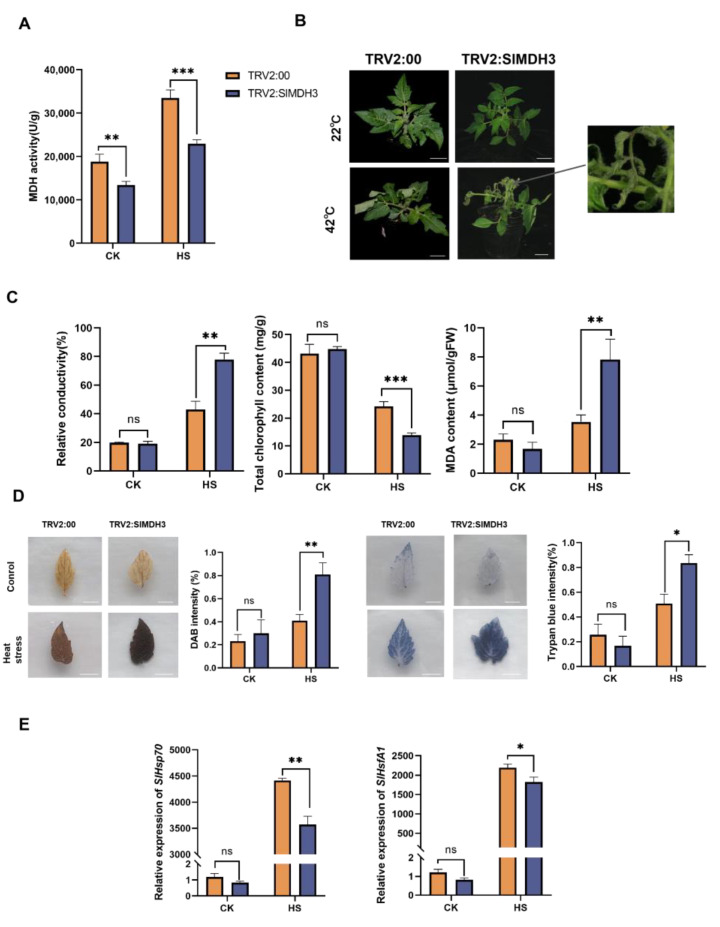
*SlMDH3* silencing increases tomato’s sensitivity to heat stress. (**A**) MDH activity in TRV2:00 and TRV2:SlMDH3 plants. (**B**) Phenotypes of tomato seedlings. (**C**) Relative conductivity, total chlorophyll content, and MDA content in TRV2:00 and TRV2:SlMDH3 plants. (**D**) Accumulation of dead cells (stained using Trypan blue) and H_2_O_2_ (stained using DAB) after heat stress in tomato leaves. (**E**) Changes in relative expression levels of heat tolerance marker *SlHsp70* and *SlHsfA1* after heat stress in tomato leaves. TRV2:SlMDH3, *SlMDH3*-silenced tomato plants; TRV2:00, negative control plants. *, *p* < 0.05; **, *p* < 0.01; ***, *p* < 0.001; ns, no significance.

Under normal temperature conditions, the activity of MDH in TRV2:SlMDH3 was significantly lower than that in TRV2:00. After heat treatment (42 °C for 8 h), the MDH activity increased in both plants but remained obviously lower in TRV2:SlMDH3 compared to TRV2:00 (Figure 4A), confirming the successful knockdown of *SlMDH3* in TRV2:SlMDH3 plants. After the heat stress treatment, the growing points of TRV2:SlMDH3 wilted, whereas those of the control plants TRV2:00 showed no significant wilting symptoms (Figure 4B). Additionally, both the relative conductivity and MDA content of the tomato leaves increased after heat treatment, with significantly higher levels observed in TRV2:SlMDH3 than in TRV2:00 (Figure 4C). However, after heat treatment, the total chlorophyll content of tomato leaf decreased, with markedly lower levels in TRV2:SlMDH3 than in control plants (Figure 4C). In addition, using DAB- and Trypan blue-staining methods, we measured the accumulation of H_2_O_2_ and dead cells, respectively. Under a normal temperature, there was minimal H_2_O_2_ and dead cells in tomato leaves. Following heat treatment, the accumulation of both H_2_O_2_ and dead cells increased in the leaves of both TRV2:SlMDH3 and TRV2:00, with more severe accumulation observed in the former compared to the latter (Figure 4D).

Heat shock proteins (HSPs) play important roles in facilitating protein refolding to restore cellular homeostasis and protect plants from abiotic stress [26], and heat shock transcription factors (Hsfs) are responsible for the transcriptional regulation of HSPs and serve as key components in the plant’s response to heat stress. Under normal temperature conditions, there was no significant difference in the expression levels of the heat tolerance marker genes *SlHSP70* and *SlHsfA1* in the leaves of TRV2:SlMDH3 and TRV2:00. However, after heat stress treatment, the expression level of both *SlHSP70* and *SlHsfA1* increased in the leaves of both tomato plants. Notably, in TRV2:SlMDH3 leaves, the expression levels of these two genes were significantly lower than those in TRV2:00 leaves (Figure 4E).

### 2.4. SlMDH3 Overexpression Plants Exhibit Enhanced Heat Tolerance

To further validate the impact of *SlMDH3* on the heat tolerance of tomato plants, we generated six *SlMDH3* overexpression transgenic tomato lines (OE1–OE6). The relative expression levels of *SlMDH3* in all the six *SlMDH3*-OE lines were significantly higher than that in wild-type (WT) ones (Figure S2), and the two lines, OE3 and OE6, were selected for subsequent experiments.

**Figure 5:**
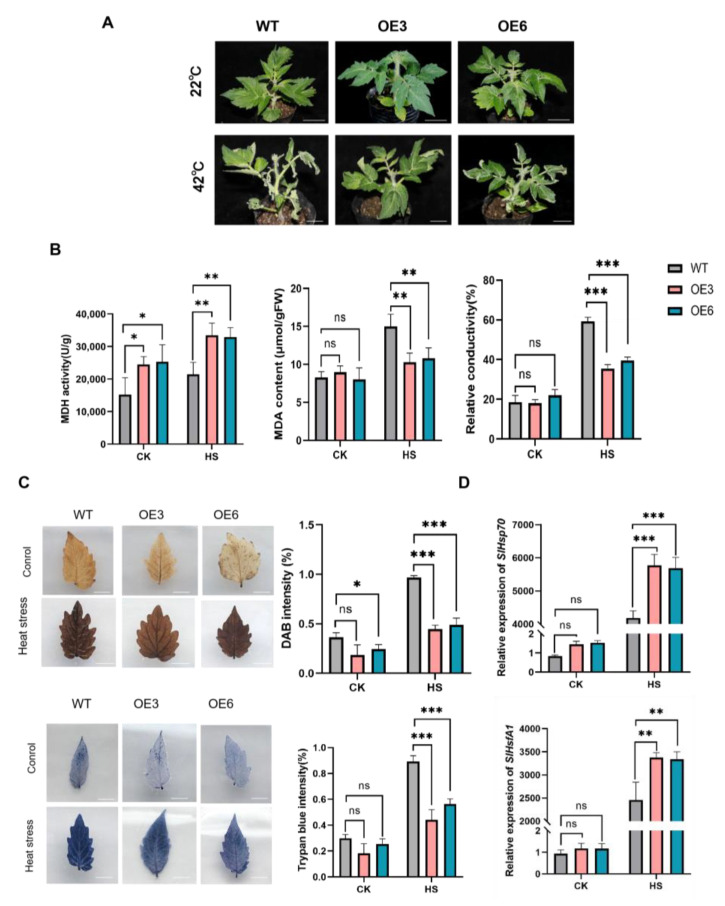
*SlMDH3* overexpression enhances tomato’s tolerance against heat stress. (**A**) Phenotypes of tomato seedlings after exposure to heat stress (42 °C for 10 h). (**B**) MDH activity, relative conductivity, and MDA content. (**C**) Accumulation of dead cells (stained using Trypan blue) and H_2_O_2_ (stained using DAB) in tomato leaves. (**D**) Changes in relative expression levels of heat tolerance marker *SlHsp70* and *SlHsfA1* after heat stress in tomato leaves. WT, wild type; OE3 and OE6, *SlMDH3* overexpression lines. *, *p* < 0.05; **, *p* < 0.01; ***, *p* < 0.001; ns, no significance.

Under normal temperature conditions, the MDH activities in the two *SlMDH3*-OE lines were notably higher than in WT. There was no significant difference in the relative conductivity, MDA level, accumulation of dead cells and H_2_O_2_, and expression levels of *SlHSP70* and *SlHsfA1* of the two lines. After heat stress treatment (42 °C for 10 h), both the WT and *SlMDH3*-OE lines displayed leaf wilting; however, the wilting degrees in the *SlMDH3*-OE lines were milder compared to WT plants (Figure 5A), and the MDH activity in the tested tomato lines increased by approximately 1.4 times; however, the activity levels in the two *SlMDH3*-OE lines were still significantly higher than that of WT (Figure 5B). Furthermore, the *SlMDH3*-OE lines showed obviously lower MDA levels (Figure 5B), higher accumulation of dead cells and H_2_O_2_ (Figure 5C), and higher expression levels of *SlHSP70* and *SlHsfA1* (Figure 5D) compared to WT.

## 3. Discussion

The aggregation of denatured protein caused by high temperature imposes toxicity on plant cells, which can be degraded through selective autophagy via the cargo recognition by autophagy receptors. Although autophagy is initially viewed as a non-selective mechanism for the bulk degradation of cytosolic components under acute stress, it has evolved into a recognized selective process known as selective autophagy [15]. This paradigm shift has unveiled specific mechanisms where pivotal proteins such as Autophagy-Related Protein8 (ATG8) play indispensable roles in both bulk and selective autophagy [27]. As an autophagy receptor, ATI1 has been reported to play important roles in plants’ responses to carbon starvation [17,18,28], salt stress [18], viral infection [18], and exogenous phytohormone [17] by targeting various cargoes for their degradation in vacuole.

In our previous study, we discovered that SlATI1 plays a positive role in regulating the heat tolerance of tomato. To explore the underlying mechanism, in this study, we identified SlMDH3 as an SlATI1-interacting protein (Figure 1) and found that the expression of *SlMDH3* was induced by heat treatment (Figure 3). To further elucidate the involvement of *SlMDH3* in the tomato’s response to heat stress, we developed *SlMDH3*-silenced plants and *SlMDH3-*overexpressed lines. Heat stress often induces alterations in malondialdehyde (MDA) levels, serving as a marker for lipid peroxidation and oxidative stress [29]. Plant cell membranes play a pivotal role in maintaining the cell’s microenvironment and normal metabolic activities. Under optimal conditions, cell membranes selectively permeate substances. However, exposure to adversities such as extreme temperatures, drought, salinity, and pathogenic infections can damage the cell membrane, leading to increased permeability, electrolyte leakage, and heightened conductivity. Numerous studies have evidenced that chlorophyll biosynthesis diminishes under high-temperature stress [30], thereby impairing chlorophyll biosynthesis in plastids [31]. Thus, we assessed pertinent physiological parameters under various environmental conditions (Figure 4 and Figure 5). Our findings revealed a notable decrease in chlorophyll content in silenced plants compared to the wild-type (WT) plants. However, conductivity and MDA levels exhibited an inverse trend, suggesting that gene silencing potentially exacerbates tomato plants’ susceptibility to heat stress. Additionally, we quantified dead cells in plant samples using Trypan Blue staining and evaluated hydrogen peroxide accumulation in leaves through DAB staining to assess oxidative stress levels. As anticipated, the silenced genotype displayed more intense staining in both methods compared to the WT leaves, indicating that gene silencing heightens the plant’s sensitivity to heat stress to some extent. Conversely, overexpression of the gene resulted in a complete reversal of all indicators (Figure 5), suggesting a potential role of this gene in enhancing tomato plants’ resistance to heat stress.

A high temperature leads to the accumulation of ROS in plant cells, disturbing the redox homeostasis and causing protein denaturation, cytomembrane damage, and even cell death [32]. MDH catalyzes the conversion of malate to oxaloacetate, coupled with the conversion of NAD^+^ to NADH, which is important for plants to produce reductant equivalents in response to developmental requirements and changing environments [33]. In our study, compared to normal conditions, after heat stress treatment, we observed the accumulation of ROS and dead cells, peroxidation of membrane lipids, and damage to the cytomembrane’s integrity in tomato leaves (Figure 4 and Figure 5). All these adverse effects imposed by heat stress were exacerbated by *SlMDH3* silencing but mitigated by *SlMDH3* overexpression, suggesting that *SlMDH3* contributes to tomato’s heat tolerance by controlling redox homeostasis. Kandoi also observed that the overexpression of maize chloroplast NADP-MDH in Arabidopsis enhanced salt tolerance by promoting the malate valve to maintain redox homeostasis inside and outside the plastid [21]. Wang suggested that the apple cytosolic MDH conferred higher tolerance to cold and salt stresses in transgenic apple plants by producing more reductive redox states [34]. However, Wu found that Arabidopsis mitochondrial MDH2 negatively regulated tolerance to Cd stress by modulating ROS levels [35]. Nan reported that rice chloroplast MDH1 negatively regulated tolerance to salt stress by affecting vitamin the B6 content and ROS accumulation [36]. These contradictory results may be related to the subcellular locations and functional diversity of MDHs, and further investigation is needed.

The subcellular translocation of proteins is a countermeasure employed by plants in response to heat stress, altering their interaction with other molecular components [37]. Upon exposure to heat stress, the Arabidopsis glycolytic enzyme glyceraldehyde-3-phosphate dehydrogenase (AtGAPC) and rice NAC transcription OsNTL3 (NTM1-like3) relocate from the cytoplasm to the nucleus, enabling them to transmit the heat signal and trigger downstream heat responses [38,39]. In this study, we observed the co-localization of SlMDH3 with SlATI1 (Figure 3B), suggesting that SlMDH3 may function together with SlATI1 in tomato to combat heat stress. ATI1, as an autophagy receptor protein, interacts with the autophagy core protein ATG8. When participating in autophagy, it typically first forms a distinct ATI body before being fused or engulfed by autophagosomes. Interestingly, the punctate co-localization of SlMDH3 and SlATI1 that we observed appears to be dynamic, suggesting the possibility of ATI1 involvement in the formation of ATI bodies and its participation in autophagy. However, whether these structures do, indeed, represent autophagosomes and whether *SlMDH3* is involved in the autophagy process remains unclear. Therefore, further research is needed to delve deeper into the relationship between them in future studies.

In summary, we identified the SlATI1 interaction protein SlMDH3, which plays an active role in regulating tomato’s tolerance to heat stress

## 4. Methods and Materials

### 4.1. Plant Materials and Growth Conditions

The tomato (*Solanum lycopersicum* L. cv. Micro-Tom) and tobacco (*Nicotiana benthamiana*) used in this study were grown with a thermoperiod of 16 h light/8 h darkness at 22 °C/20 °C and 22 °C/18 °C, respectively. The light intensity was 200 μmol·m^−2^·s^−1^, and the relative humidity was approximately 70%.

### 4.2. LC-MS/MS (Liquid Chromatography with Tandem Mass Spectrometry) Assay

The coding sequence (CDS) of *SlATI1* was cloned into pART27 with a GFP-tag using homologous recombination Assembly Mix (638947, TaKaRa, Kyoto, Japan). The resulting expression vector, pART27-GFP-SlATI1, was then transformed into an *Agrobacterium* strain GV3101. Overnight-cultured Agrobacterial suspension (OD_600_ = 0.8) was injected into tobacco leaves. The inoculated plants were grown for 3 d and then subjected to a 3 h treatment of 42 °C, and the untreated plants were used as control. The total protein was extracted with GTEN buffer as described by Tameling and Baulcombe [40] and purified following the method of Fan [41]. The purified protein was subjected to enzymatic hydrolysis and desalination and then analyzed using LC-MS/MS (API 2000, AB Sciex, Framingham, MA, USA). The number of peptides and the peptide spectrum matches (PSMs), as well as the protein false discovery rate (FDR) Confidence: Combined, were used to screen for candidate proteins of interest. The final candidate proteins were further filtered by comparing the treated and control groups.

### 4.3. Yeast Two-Hybrid (Y2H) Assay

The CDS of *SlMDH3* and *SlATI1* were individually cloned into the pPR3-N vector and pBT3-N vector, and the recombinant plasmids, pBT3-N-SlATI1 and pPR3-N-SlMDH3, were co-transformed into Y2H Gold yeast cells according to the manual of the Saccharomyces cerevisiae transformation kit (PT1199, Pytbio, Wuhan, China). The transformed yeast cells were grown on SD/Leu/-Trp and SD/-Ade/-Hiss/-Leu/-Trp medium.

### 4.4. Co-IP (Coimmunoprecipitation) Assay

The CDS of *SlMDH3* or *SlATI1* were cloned into pART27-GFP and pART27-Myc, respectively. The resulting vector was then transformed into the *Agrobacterium* strain GV3101. The bacterial suspension was injected into tobacco leaves, which were cultured for 3 d. Leaves of transformed tobaccos were collected, and then, total proteins were extracted as described above. Two protein pairs, pART27-Myc-SlATI1 + pART27-GFP and pART27-GFP + pART27-Myc, were used as negative controls. Samples were ground in liquid nitrogen and homogenized in immunoprecipitation (IP) buffer (10 mM HEPES, pH 7.4, 150 mM NaCl, 2 mM EDTA, and 10% glycerol) with 0.3% Triton X-100. A portion of the total lysis solution was reserved as the input, and the remaining lysate was incubated with anti-GFP Affinity beads (SA070001, Smart-Lifesciences, Changzhou, China) for 2 h at 4 °C. The beads were then collected and washed three times with balanced solution (50 mM Tris, 500 mM NaCl, pH 7.4) and subsequently loaded with loading buffer before being incubated at 95 °C for 5 min. IP products and the input samples were separated using 12% SDS-PAGE, and the target proteins were detected by Western blot using anti-GFP or anti-Myc antibodies.

### 4.5. Split Luciferase Assay

The CDS without a termination codon of *SlMDH3* and the full CDS with the termination codon of *SlATI1* were cloned into the expression vector pCAMBIA1300 to generate the expression vectors pCAMBIA1300-NLuc-SlMDH3 and pCAMBIA1300-SlATI1-CLuc, respectively, which were then individually transformed into *Agrobacterium* strains GV3101. The bacterial suspensions were co-injected into tobacco leaf and cultured for 3 days, and the fluorescence was observed according to the protocol described by Chen [42].

### 4.6. BiFC Assay

The CDSs of *SlATI1* and *SlMDH3* were cloned into the pSPYNE and pSPYCE vector, respectively, and the resulting constructs were transformed into the *Agrobacterium* strain GV3101. The bacterial suspensions were co-injected into the lower epidermis of tobacco leaves, which was then cultured in darkness for 3 d. The observation of YFP fluorescence was executed with a Laser Scanning Confocal Microscope (FV3000, Olympus, Japan) at excitation/emission (ex/em) 514/530–555 nm, and the tobacco co-transformed with CE-SlMDH3 + NE was used as the negative control.

### 4.7. Subcellular Localization of SlMDH3

The vector, pART27-GFP-SlMDH3, was transformed into the *Agrobacterium* strain GV3101. The empty vector, pART27, served as the control. The resulting Agrobacteria solution was injected into the back surface of 40-day-old tobacco leaves, and the plants were incubated overnight at 22 °C in darkness and then transferred to normal conditions for 2–3 d. The expression vectors pART27-GFP-SlMDH3 and pART27-mCherry-SlATI1 were co-transformed into tobacco leaf for co-localization analysis. The fluorescence signal was observed using Laser Scanning Confocal Microscope (FV3000, Olympus, Tokyo, Japan).

### 4.8. Analysis of SlMDH3 Expression of Tomato Seedlings during Heat Stress

Tomato seedlings at the 5- to 6-leaf stage were subjected to a 42 °C treatment. Leaves were sampled at 0 h, 1 h, 2 h, 4 h, 6 h, 8 h, 10 h, and 12 h post-treatment, immediately frozen in liquid nitrogen, and stored at −80 °C for total RNA extraction.

### 4.9. Generation of SlMDH3-Silenced Tomato Plants

The *SlMDH3*-silenced tomato plants were generated using virus-induced gene silencing (VIGS) as described [43]. The gene-silencing fragment of *SlMDH3* was selected using the SGN VIGS Tool (https://vigs.solgenomics.net/ accessed on 19 February 2023.) and amplified using the primer pair TRV2:SlMDH3-F/R (Table S2). The PCR product was ligated to pTRV2:00 to construct the gene-silencing vector pTRV2:SlMDH3. Tomato seedlings transiently transformed with pTRV2:SlMDH3 were used as *SlMDH3*-silenced plants (TRV2:SlMDH3), and those with pTRV2:SlPDS and pTRV2:00 were used as positive and negative controls, respectively (TRV2:SlPDS and TRV2:00). Upon observation of albino symptoms in the leaves of TRV2:SlPDS plants, the relative expression levels of *SlMDH3* in TRV2:SlMDH3 and TRV2:00 were tested, and the silencing efficiency of *SlMDH3* was calculated. Tomato plants with silencing efficiencies greater than 70% were chosen for subsequent experiments [44].

### 4.10. Generation of Tomato Lines with SlMDH3 Overexpression

Tomato cotyledons were used as explants for the generation of *SlMDH3* overexpression (*SlMDH3*-OE) lines. The healthy cotyledons were inoculated with the *Agrobacterium* strain GV3101, transformed with pART27-GFP-SlMDH3. The expression level of *SlMDH3* in the tomato *SlMDH3*-OE lines was assessed using a qRT-PCR assay with the primer pair qSlMDH3-F/R (Table S2).

### 4.11. Experimental Treatments

For heat stress treatment, tomato seedlings were subjected to a high temperature of 42 °C treatment for 8 h (for TRV2:00 and TRV2:SlMDH3) or 10 h (for WT and *SlMDH3*-OE lines). Following the treatments, the phenotypes of tomato plants were recorded through photography, and tomato leaves were sampled for the measurements of physiological indices and gene expressions. Each treatment was replicated three times, with each replicate comprising six tomato seedlings.

### 4.12. Total RNA Extraction, cDNA Synthesis, and qRT-PCR Analysis

Total RNA was isolated from tomato leaves using the Trizol kit (Invitrogen, Waltham, MA, USA), and the synthesis of first-strand cDNA was conducted using the PrimeScript Kit (TaKaRa, Kusatsu, Japan). The qRT-PCR assay was carried out using SYBR Premix Ex Taq II (TaKaRa, Japan). The relative gene expression levels were calculated following the method of 2^−ΔΔCT^ [45], in which SlUbi3 served as the internal control. All the sequences of primer pairs are listed in Table S2.

### 4.13. Determination of Physiological Indexes

The MDH enzyme activity was determined using NAD-MDH Activity Detection Kit (Solarbio BC1040) following the manufacturer’s instructions. The lipid peroxidation in cell membranes of tomato leaf discs were quantified by measuring the MDA content as previously described by Luo [46]. The total chlorophyll content was assessed using a spectrophotometric method following extraction into 80% acetone (*v*/*v*) [47]. The relative conductivity of tomato leaves was measured according to the method of Luo [46]. The accumulation of H_2_O_2_ and dead cells was observed using the methods of Zimmermann [48] and Wang [49], respectively. All experiments were performed with three biological replicates.

### 4.14. Statistical Analysis

The experimental data were plotted using Graphpad 9.5 software, and the significant difference in data between different treatments was analyzed using *t*-test in SPSS 23.0 software at the 0.05, 0.01, and 0.001 levels.

## Data Availability

The data presented in this study are available on request from the corresponding author.

## Supplementary Materials

The supporting information can be downloaded at: https://www.mdpi.com/article/10.3390/ijms25137000/s1.
