# Supplementary material for: SlMDH3 Interacts with Autophagy Receptor Protein SlATI1 and Positively Regulates Tomato Heat Tolerance
Source: Int J Mol Sci. 2024 Jun 26;25(13):7000. doi: 10.3390/ijms25137000 (PMC11241746; doi:10.3390/ijms25137000)
Supplement: Supplementary file 1 [file ijms-25-07000-s001.zip › ijms-3049415-supplementary.pdf]

Supplementary Materials

Journal Name:

International Journal Of Molecular Sciences

Article Title:

SlMDH3 interacts with autophagy receptor protein SlATI1 and positively regulates tomato heat tolerance

Sitian Wang[1], Li Zhang[1], Linyang Zhang[1], Kang Yong[1], Tao Chen[1], Lijun Cao[2,3], Minghui Lu[1*]

[1]College of Horticulture, Northwest A&F University, Yangling, 712100, China
[2]Department of Biology, Box 90338, Duke University, Durham, NC 27708, United States
[3]Howard Hughes Medical Institute, Duke University, Durham, NC 27708, United States
*Corresponding Author
Email: xnjacklu@nwsuaf.edu.cn
Tel: +86-29-87082220; Fax: +86-29-87082613

SUPPLEMENTARY MATERIALS

Table S1. Candidates of SlATI1-interacting protein identified with LC-MS assay.

Table S2. Information of PCR primers.

Figure S1.Phenotype and silencing efficiency of *SlMDH3*-silenced tomato seedlings. TRV2:SlMDH3, *SlMDH3*-silenced tomato plants; TRV2:00, the negative control; TRV2:SlPDS, the positive control. **, p<0.01.

Figure S2. *SlMDH3*-overexpression lines. **, p<0.01; ***, p<0.001.

Table S1. Candidates of SlATI1-interacting protein identified with LC-MS/MS assay.

Protien ID	Protein name	Predicted funtions
Niben101Scf01350g03009.1	D-3-phosphoglycerate dehydrogenase	Encode the 3-phosphoglycerate dehydrogenase, which is crucial for embryonic and pollen development.
Niben101Scf02004g00032.1 sp	peroxisomal NAD-malate dehydrogenase 1	Encoding peroxisome NAD malate dehydrogenase, which participates in the process of converting fatty acyl CoA to acetyl CoA by providing NAD to fatty acids β Oxidation.
Niben101Scf12308g00007.1 sp	ATP synthase alpha/beta family protein	Encoding mitochondrial ATP synthase β subunit .
Niben101Scf04329g06015.1 sp	chloroplast signal recognition particle 54 kDa subunit	Chloroplast signal recognition particle 54KDA subunit
Niben101Scf06910g05004.1 sp	delta1-pyrroline-5-carboxylate synthase 1	Coding δ- 1-Pyrroline-5-Carboxylate Synthase is a rate limiting enzyme that catalyzes the biosynthesis of proline.
Niben101Scf04886g05003.1 sp	heat shock cognate protein 70-1	A member of the heat shock protein 70 family that encodes heat shock proteins.

Table S2. Information of PCR primers.

Primer purpose	Primer Name	Sequence(5'-3')
Verification of interactive relationship	pT3-N-SlMDH3-F	CGCAGAGTGGCCATTACGGCCATGCAGCGAAGTGCTGAACGAAT
	pT3-N-SlMDH3-R	TCTCGAGAGGCCGAGGCGGCCTCATTTCTTGACAAACCCGACGC
	pPR3-N-SlATI1-F	CAACGCAGAGTGGCCATTACGGCCATGGCAAGTAATGAGGAAGGTG
	pPR3-N-SlATI1-R	GAATTCTCGAGAGGCCGAGGCGGCCTCAATGTTGAGCTGCTCCACTCCCTC
	CE-SlMDH3-F	TGGCGCGCCACTAGTGGATCCATGATGCAGCGAAGTGCTGAACGAAT
	CE-SlMDH3-R	GTACATCCCGGGAGCGGTACCTCATTTCTTGACAAACCCGACGC
	NE-SlATI1-F	CCCAGGCCTACTAGTGGATCCATGGCAAGTAATGAGGAAGGTG
	NE-SlATI1-R	CTCCTACCCGGGAGCGGTACCATGTTGAGCTGCTCCACTCCCTC
	NLuc-SlMDH3-F	ACGGGGGACGAGCTCGGTACCATGCAGCGAAGTGCTGAACGAAT
	NLuc-SlMDH3-F	CGCGTACGAGATCTGGTCGACTTTCTTGACAAACCCGACGCCCT
	SlATI1-CLuc-F	TACGCGTCCCGGGGCGGTACCATGGCAAGTAATGAGGAAGGTGA
	SlATI1-CLuc-R	ACGAAAGCTCTGCAGGTCGACTCAATGTTGAGCTGCTCCACTCC
	pART27-GFP-SlATI1-F	GATGAACTATACAAAGAATTCATGGCAAGTAATGAGGAAGGTG
	pART27-GFP-SlATI1-R	CAGGACTCTAGATTAGGTACCTCAATGTTGAGCTGCTCCAC
	pART27-Myc-SlATI1-F	TTTGGAGAGGACACGCTCGAGATGGCAAGTAATGAGGAAGGTG
	pART27-Myc-SlATI1-R	GTTAATTAACCCCATCTCGAGATGTTGAGCTGCTCCACTCCCTC
Construction of Subcellular localization / Overexpression vector	part27-GFP-SlMDH3-F	GATGAACTATACAAAGAATTCATGCAGCGAAGTGCTGAACGAAT
	part27-GFP-SlMDH3-R	CAGGACTCTAGATTAGGTACCTCATTTCTTGACAAACCCGACGC
	part27-mCherry-SlATI1-F	GACGAGCTGTACAAGGAATTCATGGCAAGTAATGAGGAAGGTG
	part27-mCherry-SlATI1-R	CAGGACTCTAGATTAGGTACCTCAATGTTGAGCTGCTCCAC
Analysis of gene expression	qSlMDH3-F	GATGCTCTCACTGGCATGGA
	qSlMDH3-R	AAAGGTGCCAGCCATCTTGA
	qSlHSP70-F	GACCAAGGGAACAGGACGAC
	qSlHSP70-R	GACGCTTGGCATCAAACACG
	qSlHSfA1-F	GAAAGCTTGCACTGACGCA
	qSlHSfA1-R	GGGGGAGTTCATTGCTCTACA
	qSlubi3-F	TCGTAAGGAGTGCCCTAATGCTGA
	q-Slubi3-R	CAATCGCCTCCAGCCTTGTTGTAA
Construction of gene-silencing vector	TRV2:SlPDS-F	GTGAGTAAGGTTACCGAATTCGCTCGAGGTCGTCTTCTTTGGGA
	TRV2:SlPDS-R	GAGACGCGTGAGCTCGGTACCAGTTGGGCGCGGAGAAGCAC
	TRV2:SlMDH3-F	GTGAGTAAGGTTACCGAATTCGGCCATTGTCAATATAATTAG
	TRV2:SlMDH3-R	GAGACGCGTGAGCTCGGTACCGAACAAGGAGGCTTAATCTGG

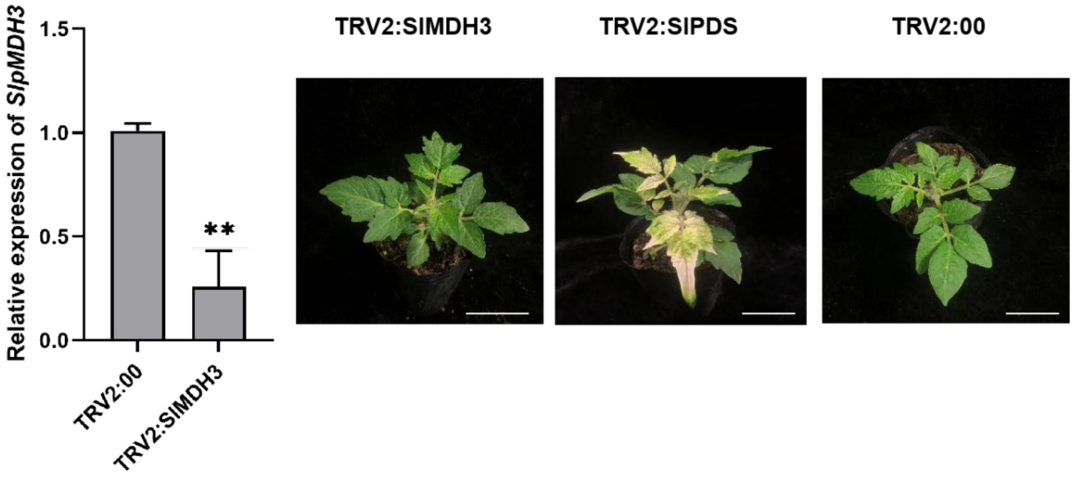

Figure S1.Phenotype and silencing efficiency of *SlMDH3*-silenced tomato seedlings. TRV2:SlMDH3, *SlMDH3*-silenced tomato plants; TRV2:00, the negative control; TRV2:SlPDS, the positive control. **, p<0.01.

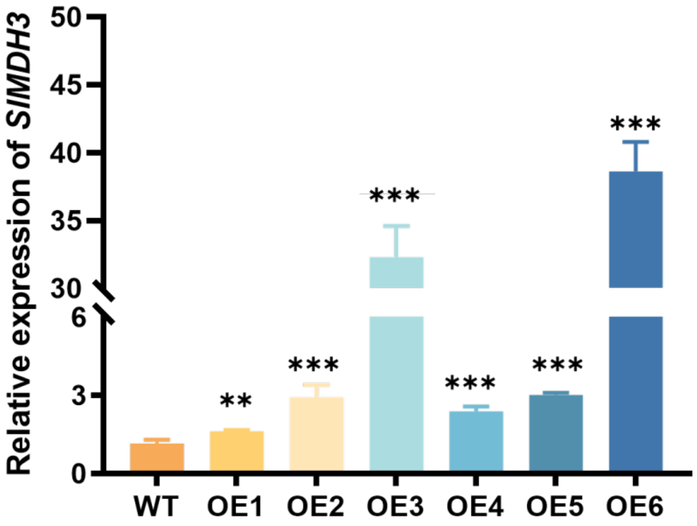

Figure S2. *SlMDH3*-overexpression lines. **, p<0.01; ***, p<0.001.
